# Sorrow Kills

**Published:** 1871-05

**Authors:** 


### SORROW KILLS.

Not very long since it was announced in the papers, far and near, that Robert Browning, the poet, was in a condition little short of the paradise of health. His own pen certified the truth of the statement, with some remarks attributing his good health, and his "snapping his fingers at the doctors," to certain modes of life, which, to an intelligent mind, seemed questionable as to their wisdom. Well, he didn't snap his fingers long, for now, at the age of fifty-eight years, when he should be still in his manly prime, in the very vigor of physical and mental strength, it is written of him that since his wife's death he has grown very old in appearance, and, judged by his face, he would be taken to be seventy years of age.

### ROOT, PIG, OR DIE,

Is one of the wisest and most benevolent of all the principles involved in human creation ; and the Maker of us all has so arranged it that among the greatest and most constant pleasures of life should be eating and drinking and dress ; and that toil, for the gratification of these, should become a pleasure and a happiness.

### USEFUL INDUSTRIES

Are the grand panaceas for human sorrow ; they are the cure-alls of the heart. If you are sad for an hour, go and do something ; if any heaviness weighs down your heart day after day, the best thing you can do is to embark in some enterprise with risk or responsibility enough about it to force out all your mental and physical vigor. Brooding over any sorrow is a weakness unworthy of a man. Every intelligent mind ought to accustom itself to stand in its lot when the early and the minor troubles of life begin to show themselves, and meet them face to face, thus accustoming itself to the conflict, which becomes more and more severe inevitably as years come on ; then there will rise by degrees a skill and a bravery in meeting them, which makes the battling with them not without its satisfactions.

Indulgence in grief does no good; it gradually grows upon the mind, and often unhinges it; certainly, moodiness and melancholy, even from causes well calculated to induce them, unfit for the duties of life; and if these duties are not performed faithfully and willingly and well, we must pay the penalty. No man ever yet grandly succeeded, until he had overcome obstacles, discouragements, and disappointments; and as to the greatest characters of history, it will be found that difficulties gave them an inspiration, nerved them to attempts, and accomplished deeds which made them immortal.

